# A Bio-Inspired Method for the Constrained Shortest Path Problem

**DOI:** 10.1155/2014/271280

**Published:** 2014-05-14

**Authors:** Hongping Wang, Xi Lu, Xiaoge Zhang, Qing Wang, Yong Deng

**Affiliations:** ^1^School of Computer and Information Science, Southwest University, Chongqing 400715, China; ^2^School of Engineering, Vanderbilt University, Nashiville, TN 37235, USA

## Abstract

The constrained shortest path (CSP) problem has been widely used in transportation optimization, crew scheduling, network routing and so on. It is an open issue since it is a NP-hard problem. In this paper, we propose an innovative method which is based on the internal mechanism of the adaptive amoeba algorithm. The proposed method is divided into two parts. In the first part, we employ the original amoeba algorithm to solve the shortest path problem in directed networks. In the second part, we combine the *Physarum* algorithm with a bio-inspired rule to deal with the CSP. Finally, by comparing the results with other method using an examples in DCLC problem, we demonstrate the accuracy of the proposed method.

## 1. Introduction

The shortest path problem (SPP) is one of the most classic problems, which is widely used in many fields, like network optimization [1, 2], road navigation [3, 4], and so on [5–7]. The constrained shortest path (CSP) problem aims at finding the shortest path from a predetermined source node to a predetermined destination node in a directed network under the constraint of being less than or equal to a given upper limit. Such constraints on the classical shortest path problem generally result in the problem becoming NP-hard. CSP is often considered as a variant of SPP and is frequently put into practice, like tail assignment problem in aircraft scheduling [8–10], the quality of service routing in communications networks [11, 12], and so forth [13].

Strategies of solving the CSP can be classified into three main categories: Lagrangian relaxation methods, dynamic programming (DP), and path ranking methods. The first method of CSP relies on the solution to a Lagrangian dual problem and uses different approaches to close the duality gap. Handler and Zang [14] proposed a method to solve the Lagrangian dual problem. They closed the duality gap by using the *k* shortest paths algorithm, terminating with the first path satisfying the constraint. They also proposed a method which searched in a direction. The method was based on combination of the original objective and the constraint. Carlyle et al. [15] presented a new algorithm for CSP. The improvement of this method is that the generated Lagrangian function was optimized and the optimality gap was closed by enumerating near-shortest paths. Recently, Zhang et al. [16] used Lagrangian relaxation combined with an adaptive amoeba algorithm to solve the CSP. In fact, if the constraint was relaxed, it made the CSP as the classical SPP, which could be solved repeatedly by plentiful computational efforts. What is more, the efficiency of this approach relied on the effectiveness of the underlying unconstrained shortest path algorithms.

A number of previous works were based on DP. All of these methods were based on label-setting or label-correcting algorithm. Starting from the the work of Joksch [17], the approaches of node labeling were deeply investigated. Dumitrescu and Boland [18] extended this idea and solved this problem by using preprocessing techniques. They [18] showed how the performance of the label-setting method could be further improved by making use of all Lagrange multiplier information collected in a Lagrangian relaxation's first step. Recently, Likhyani and Bedathur [19] developed SkIt index structure, which supported a wide range of label constraints on paths, and returned an estimation of the shortest path that satisfied the constraints. However, all of these methods mentioned above had a disadvantage: they might fail to well-estimate very large networks due to the “curse of dimensionality.” As a result, it could not apply to real world with a large size of nodes.

A path ranking method was presented by Handler and Zang [14] by *k* shortest paths algorithm. Santos et al. [20] extended this idea by improving the way of sorting the *k* shortest paths. Its direction was based on the relative tightness of the constraint. Jia and Varaiya [21] presented two new methods based on the *k*-shortest-path approach. These heuristic methods, where one is centralized and the other is distributed, were both polynomial. In numerical experiments the proposed algorithms almost found the optimal paths. The main drawback of this method is that large value for *k* is required before a feasible solution of CSP is found.

Recently, a slime mold called* Physarum polycephalum* has attracted many investigators [22–25]. The body of the plasmodium of* Physarum polycephalum *contained a network of tubular elements by means of which nutrients and chemical signals circulated through the organism in an effective manner [26, 27]. Since the tubes disassembled and reassembled within a period of a few hours in response to external conditions, this organism was very useful for studying the function and dynamics of natural adaptive networks [28–30]. It was used to find the shortest path [31–33], solve the amaze [34, 35], optimize the networks [36, 37], and structure the road networks [38–40] and other fields [41–45]. Due to the uncertainty in real application [46–53], this algorithm is also applied to solve shortest path under uncertain environment [54, 55].

Inspired by the intelligence of the amoeba, we combine the* Physarum* algorithm with a bioinspired rule to deal with the CSP. The main idea behind the approach is to employ* Physarum* algorithm to find the shortest path. Then a check procedure is processed to ensure it satisfies the constraint. Once it does not meet the constraint, a penalty function is conducted. This procedure will go on executing until the path satisfying the constraint is found.

The paper is organized as follows. In Section 2, the basic theories, including CSP and the* Physarum* model, are introduced. In Section 3, the bioinspired method which is based on amoeba algorithm for CSP is proposed. In Section 4, an example is used to illustrate the proposed method. In comparison with other methods, the validity of the proposed method is demonstrated. In Section 5, the conclusion is given.

## 2. Basic Theories

In this section, we will introduce some basic theories, including the constrained shortest path problem and* Physarum polycephalum* model.

### 2.1. The Constrained Shortest Path Problem

Let *G* = (*V*, *E*) be a directed network, where *V* = 1,…, *n* is the set of nodes and *E* = (*i*, *j*) : *i*, *j* ∈ *N*, *i* ≠ *j*, is the set of directed edges. Each edge has two nonnegative weights *c*_*ij*_ and *t*_*ij*_. *c*_*ij*_ and *t*_*ij*_ represent the generalized cost and the constrained variable, respectively. The CSP can be expressed by the following mathematical formulation:

(1) $$\begin{array}{c} \min\underset{ij}{\sum }c_{ij}x_{ij}, \end{array}$$

subject to

(2) $$\begin{array}{c} \underset{ij}{\sum }x_{ij}-\underset{j,i}{\sum }x_{ji}=\{\begin{array}{cc} 1, & \text{for}\;\;j=1,\\ -1, & \text{for}\;\;j=2,\\ 0, & \text{for}\;\;\text{otherwise}, \end{array} \end{array}$$

(3) $$\begin{array}{c} \underset{ij}{\sum }t_{ij}x_{ij}⩽T, \end{array}$$

(4) $$\begin{array}{c} x_{ij}\in \left\{0,1\right\},\;\forall i,j, \end{array}$$

where *x*_*ij*_ is binary variable, which is defined as follows:

(5) $$\begin{array}{c} x_{ij}=\{\begin{array}{cc} 1, & \text{if}\;\;x_{ij}\;\;\text{is}\;\;\text{in}\;\;\text{optimal}\;\;\text{path},\\ 0, & \text{others}. \end{array} \end{array}$$

The parameter *T* indicates the maximum value allowed for the sum of *t*_*ij*_. Equations (1), (2), and (4) define the shortest problem. The constraint (3) causes the CSP to be an NP-hard problem.

### 2.2. *Physarum polycephalum* Model

*Physarum polycephalum* is a single-celled amoeboid organism that can form a dynamic tubular network to link the discovered food sources during foraging. The physiological mechanism behind the phenomenon is that tubes thicken in a given direction when the flux through it persists in that direction for a certain time. By this mechanism, a mathematical model for path finding has been constructed to describe the adaptive dynamics of tubular network. In what follows, we will give a brief introduction to this path finding model [56].

Suppose the shape of the network formed by the* Physarum* is represented by a graph; the edge and the junction in the graph can be treated as a plasmodial tube and the node, respectively. Two special nodes labeled as *N*_1_ and *N*_2_ act as the source node and sink node, respectively. The other nodes are labeled as *N*_3_, *N*_4_, *N*_5_, *N*_6_, and so forth. The edge between nodes *N*_*i*_ and *N*_*j*_ is expressed as *M*_*ij*_. The parameter *Q*_*ij*_ denotes the flux through tube *M*_*ij*_ from node *N*_*i*_ to *N*_*j*_. Assuming the flow along the tube is an approximate Poiseuille flow, the flux *Q*_*ij*_ can be expressed as

(6) $$\begin{array}{c} Q_{ij}=\frac{D_{ij}}{L_{ij}}\left(p_{i}-p_{j}\right), \end{array}$$

where *p*_*i*_ is the pressure at the node *N*_*i*_, *D*_*ij*_ is the conductivity of the tube *M*_*ij*_, and *L*_*ij*_ is its length.

By considering that the inflow and outflow must be balanced, we have

(7) $$\begin{array}{c} \sum Q_{ij}=0\;\left(j\neq 1,2\right). \end{array}$$

As for the source node *N*_1_ and the sink node *N*_2_ the following two equations hold:

(8) $$\begin{array}{c} \underset{i}{\sum }Q_{i1}+I_{0}=0,\\ \underset{i}{\sum }Q_{i2}-I_{0}=0, \end{array}$$

where *I*_0_ is the flux flowing from the source node, which is set as a constant in the model.

Then the network Poisson equation for the pressure can be derived from (6)–(8) as follows:

(9) $$\begin{array}{c} \underset{i}{\sum }\frac{D_{ij}}{L_{ij}}\left(p_{i}-p_{j}\right)=\{\begin{array}{cc} -1, & \text{for}\;\;j=1,\\ +1, & \text{for}\;\;j=2,\\ 0, & \text{otherwise}. \end{array} \end{array}$$

By setting *p*_2_ = 0 as a basic pressure level, all *p*_*i*_ can be determined by solving (9) and *Q*_*ij*_ can also be obtained according to (6).

With the mechanism between the flux and tube thickness (described as the conductivity of the tube), the adaptation equation for conductivity is constructed as follows:

(10) $$\begin{array}{c} \frac{d}{dt}D_{ij}=f\left(\left|Q_{ij}\right|\right)-rD_{ij}, \end{array}$$

where *r* is a decay rate of the tube. It can be obtained from the equation that the conductivity will finally vanish if there is no flux along the edge, while it is enhanced by the flux. The *f* is monotonically increasing continuous function satisfying *f*(0) = 0.

## 3. Proposed Method

The original amoeba algorithm can only find the shortest path in undirected networks. In order to solve the CSP in directed networks, we have to solve two problems. The first one is how to find the shortest path in directed networks. The second one is how to efficiently solve CSP by modified amoeba algorithm.

### 3.1. Modified Amoeba Model for the Shortest Path Problem in Directed Networks

Let *G* = (*N*, *E*, *W*) be a directed network, where *N* represents the set of *n* nodes, *E* represents the set of directed edges, and *W* represents the weight set of *E*. We suppose that there is only one direction between node *i* and node *j* in the network *G*. Assume node *s* and node *t* are source node and sink node, respectively. The shortest path problem in the network can be defined as how to find a path from node *s* to node *t*, which only consists of directed edges of *E*, with the minimum sum of weights on the edges.

In the original amoeba model, every arc is bidirectional. It means that the flux can flow from node *i* to node *j*. They can also flow in the opposite direction. It is no different for original amoeba model, while the direction should be strictly distinguished in the directed networks. In particular, there is only one direction between two nodes in network *G*. It means that if the flux can flow from node *i* to node *j*, they cannot flow in the opposite direction. In order to take the factor of direction into account, each edge is regarded as two tubes with opposite direction and equal weight. Here, we assume the weight represents the length between two nodes represented by *L*. If there is a link between nodes *i* and *j*, then there are two tubes with equal length *L*_*ij*_ and *L*_*ji*_, respectively, denoting two opposite directions from node *i* to node *j* and from node *j* to node *i*. The initial value of conductivity along with each tube is used to imply whether the tube is accessible or not. If arbitrary tube satisfies *L*_*ij*_ ∈ *E*, then the corresponding initial value of conductivity *D*_*ij*_ is assigned an arbitrary value among [0.5, 1]. Besides, *D*_*ji*_ is assigned as 0. The matrix *D* implies not only the conductivity but also the direction of each edge. By this way, the factor of direction is considered. In order to make original amoeba model that can adapt the directed networks, (9) is improved as follows:

(11) $$\begin{array}{c} \underset{i}{\sum }\left(\frac{D_{ij}}{L_{ij}}+\frac{D_{ji}}{L_{ji}}\right)\left(p_{i}-p_{j}\right)=\{\begin{array}{cc} -1, & \text{for}\;\;j=s,\\ +1, & \text{for}\;\;j=t,\\ 0, & \text{otherwise}. \end{array} \end{array}$$

With the purpose of keeping the validity of conductivity, the flux along with each tube needs to be adjusted. Equation (10) is modified as follows:

(12) $$\begin{array}{c} \frac{D_{ij}^{n+1}-D_{ij}^{n}}{\delta t}=\{\begin{array}{cc} Q_{ij}-D_{ij}^{n+1}, & Q_{ij}>0,\\ -D_{ij}^{n+1}, & \text{otherwise}. \end{array} \end{array}$$

Amoeba model has a positive feedback mechanism: the higher the pressure difference is, the more the flux will be. The more the flux is, the higher the pressure difference will be. When the iteration continues, the shortest path gradually appears through this mechanism. In directed networks, the positive feedback mechanism also exists. The method for finding the shortest path in directed network is described as follows.

1. Initialize values of each parameter. According to the weight of the edge in *G*, the length of *L*_*ij*_ is initialized. According to the direction of each edge, the conductivity is initialized. Here, the initial value is assigned equally as 0.5. If there is an edge from node *i* to node *j*, *D*_*ij*_ = 0.5. Otherwise, *D*_*ij*_ = 0. The pressure of the node *i* is represented by *p*_*i*_. The basic information of this directed network is initialized.
2. According to current conductivity, the pressure of each node can be calculated using (11).
3. According to (6), we can obtain the flux of each edge.
4. According to (12), we can calculate the conductivity of the next moment.
5. If the conductivity *D*_*ij*_ of each edge is no longer changed, the termination criterion is met. The directed shortest path consists of the edges with conductivity approximately equaling 1. Otherwise, go back to Step 2 and repeat until convergence.

### 3.2. Bioinspired Method

In this section, we will introduce some nomenclatures and phenomena about amoeba. Then we will introduce the proposed method.

#### 3.2.1. Nomenclatures and Phenomena

 Segment: the tubular pseudopodia of amoeba linking node *i* to node *j*.
 Growing segment: the segments with continuous thickening.
 Potential segment: the growing segments with growing times exceeding given threshold.
 Threshold: the critical value of judging whether growing segment is potential segment.
 Important segment: the potential segments which can make up an unbroken path.
 Important path: the path which is made up by potential segments.
 Conductivity: indicating the thickness of the segment.

As the iteration continues, some segments become thicker while others degenerate. Simultaneously, the corresponding conductivity tends to increase or decrease. When the conductivity value in the current iteration is greater than the previous one, it is called thickening segment. When a segment is thickening, it means that the segment will continue to thicken. The segment becomes a growing segment. If the growing times of the growing segment exceed the threshold, it becomes a potential segment. If the potential segment belongs to the important path, it becomes an important segment. Through many trials, we find that the segment which belongs to the shortest paths continuously thickens from the beginning to the end. At the beginning, growing segments may be dispersed in the network. Then, potential segments and important segments occur. At the end, there are only important segments left, which make up a shortest path. In other words, we think some of those important segments are the final part of the shortest path and the important path is the shortest path on the great probability.

#### 3.2.2. Proposed Method

**Figure 1:**
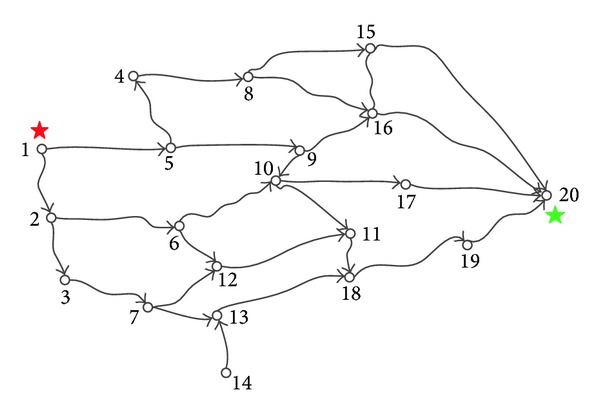
A directed transportation network with 20 nodes used in [16].

As shown in Figure 1, an example employed in [16] is used to illustrate the general flow of the proposed method. Each arc has two attributes: the length *L*_*ij*_ and the cost *C*_*ij*_. The specific values can be obtained according to Table 1. For instance, the numbers (120 and 60) along the arc between node 1 and node 2 mean the length along the arc is 120, and the cost along the arc is 60. We want to find a length shortest path from source node 1 to sink node 20 with the cost no more than 200.

Inspired by the important segment, we propose a bioinspired method based on the internal mechanism of the amoeba model. Steps are introduced in detail as follows.

 (1)  On the basis of Step 1 in Section 3.1, we need to do some extra operations as follows.
 According to the information of the given CSP, we initialize the constraint *θ*. *κ* is a parameter which is used to judge whether the growing segment is the potential segment or not. *γ* is the divisor of the punishment. When the important path does not satisfy the constraint conditions, *γ* is used to decrease the conductivity of those important segments so that the amoeba can converge to the path satisfying the constraint. Besides, we set a variable* flag* representing the end mark and assign 0, which means the end mark does not satisfy the termination criterion.
 (2)  Step 2 to Step 5 are the same as in Section 3.1.
 (3)  According to the current and the previous conductivity, the growing segments can be found and marked. The growing times of each growing segment are also saved.
 (4)  According to the saved times and the threshold *κ*, we can judge whether the growing segment exceeds the threshold. If it exceeds it, it is the potential segment. Save these potential segments.
 (5)  Judge whether these potential segments can make up a complete path from the source node to the sink node. If they can, we mark these important segments and then go to Step 6. Otherwise, go to Step 7.
 (6)  According to these important segments, the important path can be obtained. Judge whether the important path satisfies the constraint *θ*. If it can, the original data such as *D*_*ij*_, *Q*_*ij*_ is kept and makes the flag equal 1, which means the flag satisfies the termination criterion. In other words, the constrained shortest path is found. Then go to Step 7. Otherwise, do the following. The conductivities of each important segment (the segment who belongs to this important path) are decreased. The updated conductivity is equal to the original value divided by the divisor of the punishment *γ*. The marks including times and important segments are initialized.
 (7)  Check whether the termination criterion is met or not. The termination criterion is that the value of flag is 1. If it is satisfied, tubes with maximal conductivity make up the constrained shortest path. Otherwise, go to Step 2 and repeat until termination is satisfied.

**Figure 2:**
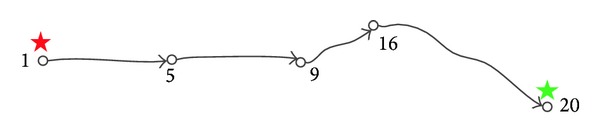
The solution.

More detailed procedures are showed in Algorithm 1. The basic idea behind this method is that we take full advantage of* Physarum* algorithm to find the path. After finding the path successfully, the check procedure is conducted to insure the path satisfies the constraint. If it does not meet the constraint, the penalty mechanism will be processed. By this way, the successive path will fade out. The program will continue to execute until the path satisfying the constraint is found. As can be seen in Algorithm 1, it is necessary for us to solve the linear equations shown in (9). The time complexity of solving the linear equations is *O*(*N* ^3^), where the |*N*| is the number of equations. In the original amoeba model, the number of equations and nodes in the network are equal. Hence, the time complexity of the bioinspired method is *O*(*N* ^3^), where the |*N*| is the number of nodes in the network. According to Figure 1, we let *s* be 1, *t* 20, and *θ* 200. Parameters *κ* and *γ* are experimental values. Here, we let them be 3 and 10, respectively. As shown in Figure 2, the solution 1-5-9-16-20 to this problem is obtained easily. The length of the path is 340 and the cost of the path is 200.

It can be noted that the solution obtained by the proposed method is the same as the result presented by Zhang et al. [16]. Both of them can find the optimal solution. Therefore, the accuracy of the presented method is demonstrated. Different from the method proposed by Zhang et al. [16], the bioinspired method combines the* Physarum* algorithm and the penalty mechanism by modifying the internal mechanism of the amoeba model while Zhang et al. [16] combine the* Physarum* algorithm with the* Lagrangian relaxation* method.

## 4. Examples

In this section, an example is introduced in order to prove the validity of the proposed method. The results are compared with the algorithm presented by Handler and Zang [14].

It is well known that the delay constrained least cost problem (DCLC) is a typical problem of CSP, which is widely used in network routing [57, 58]. The DCLC problem is to find the least cost path in a graph while keeping the path delay below a specified value. Let *G* = (*V*, *E*) be a directed network, where *V* = 1,…, *n* is the set of nodes and *E* = (*i*, *j*) : *i*, *j* ∈ *n*, *i* ≠ *j*, is the set of edges. The edge (*i*, *j*) represents the direction from node *i* to node *j*. Each edge has two nonnegative attributes: *c*_*ij*_ and *d*_*ij*_ represent cost and transmission delay from node *i* to node *j*, respectively.

Given a source node *s* ∈ *V*, a sink node *t* ∈ *V*. Δ_delay_ is the given delay constraint. Functions *D*(*p*) and *C*(*p*) represent delay and cost of the path *p*. The DCLC problem can be formulated as follows:

(13) $$\begin{array}{c} \underset{p\in P^{\prime }\left(s,t\right)}{\min}C\left(p\right). \end{array}$$

Here, *P*′(*s*, *t*) meets the following constraint:

(14) $$\begin{array}{c} \Delta_{\text{delay}}⩾D\left(p\right),\;p\in P\left(s,t\right), \end{array}$$

where *P*(*s*, *t*) represents the set of all paths from node *s* to node *t*.

**Figure 3:**
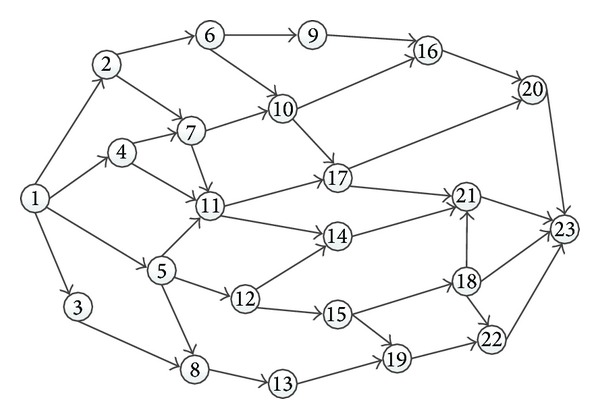
A real directed network with 23 nodes used in [59].

As shown in Figure 3, it is a real network topological structure with source node 1 and sink node 23. It was frequently an example in fuzzy shortest path problem [54, 59, 60]. The delay and cost of each link in the network are randomly generated. The costs of links are randomly generated by normal distribution with mean value equaling 10 and standard deviation equaling 3. The delays of links are randomly generated using normal distribution with mean value equaling 10 and standard deviation equaling 5. The cost and delay of each link randomly generated by normal distribution are shown in Table 2.

In order to get significative constraint, the delay constraint is got as follows [20]:

(15) $$\begin{array}{c} \Delta_{\text{delay}}=p*\left(p_{dc}-p_{ld}\right)+p_{ld}, \end{array}$$

where *p*_*ld*_ is the least delay and *p*_*dc*_ is the delay of the path which has the least cost. They can be obtained by amoeba algorithm. The parameter *p* is a problem specific ratio that measures the “tightness” of constraint (3) such that 0 < *p* < 1. The more constraining (or “tight”) the value of *T* in constraint (3) is, the lower the value of *p* will be. For simplicity, here, we let *p* = 0.1.

Here, the least delay path is 1-3-8-13-19-22-23 and its delay is 44.0553. The least cost path is 1-4-11-17-20-23 and its delay is 54.1798 and its cost is 48.7660. The constraint 45.0680 can be obtained by using (15): Δ_delay_ = 0.1∗(54.1798 − 44.0553) + 44.0553 = 45.0680.

Let *θ* be 45.0680, *κ* 2, and *γ* 30. Finally, we can get the solution: 1-3-8-13-19-22-23. The cost of the solution is 74.5885. The solution is exactly the same as the method [14] as well.  Table 3 lists these *k* shortest paths [14] and important paths.

As can be seen in Table 3, our searching order is different from Hanlder and Zangs. The reason is that the order of important path depends on the specific values of the parameters *κ* and *γ*. Generally speaking, smaller *κ* and larger *γ* lead to a bigger step when it searches for the following path in our method. Otherwise, the step will be small. It will search the alternative paths one by one. The parameters setting is in association with the scale of network and the parameter *p*. In the case of a large scale of network, different parameters result in a big difference in executing time. The problem of how to set specific parameters will be further studied in the future work.

## 5. Computational Comparisons of the Solution Algorithms

In order to demonstrate the accuracy of proposed method, a number of experiments have been conducted on different networks. In addition, the CPU run-time has been compared between our method and the method proposed by Handler and Zang [14]. The main idea of the algorithm proposed by Handler and Zang is to use the *k* shortest paths algorithm to find the next shortest path terminating with the first path satisfying the constraint. This algorithm requires solving the *k* shortest path problem. The method finding the *k* shortest loopless paths in a network proposed by Yen [61] and the Dijkstra algorithm for finding the shortest path are used to solve the *k* shortest path problem.

Sixty-five networks were randomly generated. There are 5 different networks sizes based on the number of nodes in the networks. For each network in the same size, four networks are randomly generated. For each of the four random networks, three different attributes, but the same topologies networks, are generated. These attributes are randomly generated by normal distribution.

As for the random networks, we use the BA model [62] to generate a randomly directed network. In BA model, there are two assumptions: firstly, the network is formed by the continuous addition of new vertices to the system. Therefore, the number of vertices *N* increases throughout the lifetime of the network. Secondly, when there is a new node added in the network, it tends to be connected with the vertex who has more connections.

Under this assumption, we use the following steps to construct the network.

(i) Beginning with a small network *G*_0_, it has *N*_0_ nodes and *E*_0_ edges. At each time step, we add only one new node.
(ii) Assume there are *n* nodes (*s*_1_, *s*_2_,…, *s*_*n*_). When adding a new node *s*_*n*+1_, it has *m* (⩽*N*_0_) edges linking *m* different already existing nodes in the network.
(iii) *d*_*i*_ indicates the degree of node *s*_*i*_ (1 ⩽ *i* ⩽ *n*). The linking probability *P*_*i*_ between nodes *s*_*i*_ and *s*_*n*+1_ is defined as follows:
  (16) $$\begin{array}{c} P_{i}=\frac{d_{i}}{\sum_{j=1}^{n}d_{j}}. \end{array}$$

Because the network is generated by this model, its degree distribution follows the power-law distribution with *γ*_model_ = 2.9 ± 0.1. In this paper, we let *N*_0_, *E*_0_, and *m* be equal to 3.

All parameter values are listed in Table 4.

The tests are executed on Intel(R) Core(TM) i3-2120 processor (3.30 GHz) with 2 GB of RAM under Windows Seven. Each instance is run for 3 times, and we compute the average executing time and average accuracy. Results of 65 test problems are summarized in Table 5.

After experiment, the accuracy is 95.38% in the proposed method. In all the 65 networks, there are only 3 networks we cannot get optimal solution when *κ* = 2, *γ* = 30. However, we have verified that once these two parameters are adjusted, the optimal solution can be obtained. As can be seen in Table 5, the method proposed by Handler and Zang [14] is faster than the approach proposed by this paper. However, the method combining amoeba model and penalty mechanism has three potential advantages. First of all, the CSP problem is an NP-hard problem and it is difficult for us to solve this problem. The method proposed by this paper is to contribute an innovative idea and to enrich the solution strategies for solving this NP-hard problem. Secondly, the reason why the proposed method is relatively slow is that the amoeba model in this paper is implemented in a tradition way. In fact, there are already many investigations on parallel amoeba algorithm which have dramatically decreased the computational time. For example, worthwhile works [63, 64] have been achieved. Amoeba algorithm is constantly improving and it has a great potential for the development of the bioinspired artificial intelligence. It is certain that the computational time of the proposed method will be greatly reduced if there was a sophisticatedly parallel amoeba algorithm. At the current stage, we just aim at its methodology. Finally, the proposed method is easy to combine with other algorithms. By combining with other approaches, we can take the advantages of both of them. To sum up, although the executing time of the proposed method is relatively slow, it can be improved with the implementation of the parallel amoeba algorithm. What is more, the proposed method has many advantages over the existing algorithms.

## 6. Conclusion

In this paper, we propose an innovative method to solve the CSP, which employs the internal mechanism of the amoeba model. The solution to the CSP consists of two parts. Firstly, we extend* Physarum* algorithm into the shortest path problem in directed networks. Secondly, we combine the* Physarum* algorithm with a bioinspired rule to deal with the CSP. In addition, we have shown the validity of the proposed method by comparing with other existing approaches using an example in the DCLC problem.

In the future, on the one hand, we will further investigate how to determine the associated parameters, such as *κ* and *γ*. Currently, these parameters are given according to the many experimental results. In the near future, we will try to provide theoretical analysis to these parameters and to provide an interval to these parameters. On the other hand, we will explore how to implement the proposed method in parallel environment, which will greatly reduce the executing time of the proposed method.

## Figures and Tables

**Algorithm 1:**
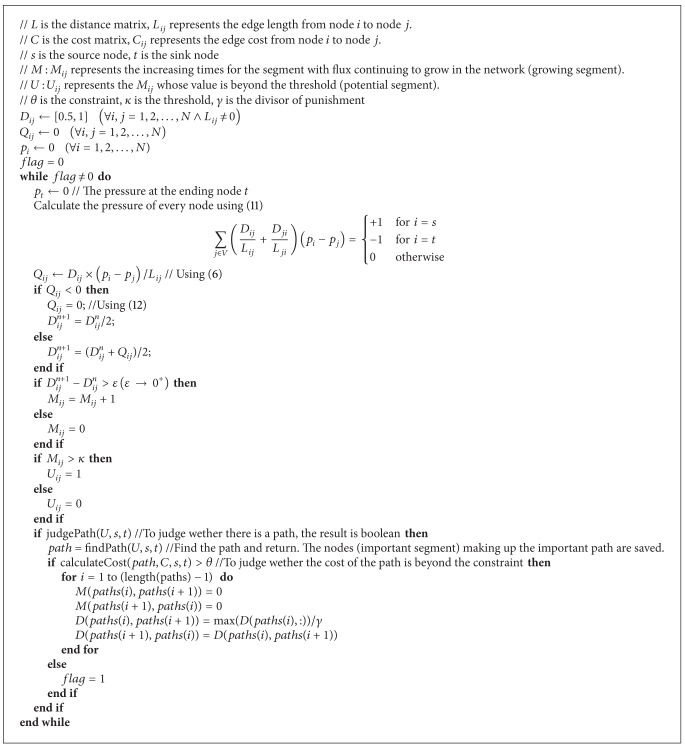
A bioinspired method for the constrained shortest path problem.

**Table 1:** Attribute value of each edge employed in [16].

Edge	Length	Cost
1 → 2	120	60
1 → 5	90	50
2 → 3	100	40
2 → 6	90	60
3 → 7	90	50
4 → 8	70	40
5 → 4	120	60
5 → 9	80	50
6 → 10	40	20
6 → 12	60	40
7 → 12	80	60
7 → 13	60	50
8 → 15	70	40
8 → 16	100	40
9 → 10	20	60
9 → 16	90	40
10 → 11	90	50
10 → 17	70	40
11 → 18	70	60
12 → 11	50	50
13 → 18	140	40
14 → 13	100	60
15 → 20	150	50
15 → 16	40	40
16 → 20	80	60
17 → 20	60	50
18 → 19	40	40
19 → 20	50	40

**Table 2:** The attributes' value associated with each edge.

Link	Cost	Delay	Edge	Cost	Delay
1 → 2	15.4922	4.3346	12 → 15	18.0059	7.1883
1 → 3	10.3278	6.9174	19 → 15	18.4855	9.0751
1 → 4	6.9264	9.2813	9 → 16	6.0788	13.7941
1 → 5	14.1113	11.0636	10 → 16	4.7168	4.0935
2 → 6	20.4833	17.6400	10 → 17	8.9175	10.1357
2 → 7	21.4486	7.7884	11 → 17	10.7635	11.7094
4 → 7	12.5672	9.6045	15 → 18	10.0545	12.9549
3 → 8	18.7087	6.8806	13 → 19	7.1035	6.8859
5 → 8	12.0780	7.2797	15 → 19	9.5816	7.9918
6 → 9	16.4558	6.3616	16 → 20	14.1424	9.7289
6 → 10	10.9584	8.1689	17 → 20	11.3280	12.6545
7 → 10	12.5739	11.7066	14 → 21	15.2885	11.3513
4 → 11	8.9370	12.9112	17 → 21	15.9805	11.7076
5 → 11	19.0253	11.5669	18 → 21	11.5145	5.7829
7 → 11	10.8697	10.4742	18 → 22	10.0890	12.2744
5 → 12	19.1114	7.4606	19 → 22	14.6551	11.5192
8 → 13	11.2672	2.6939	18 → 23	2.9196	10.9407
11 → 14	16.8435	12.3477	20 → 23	10.8112	7.6235
12 → 14	8.3291	9.7940	21 → 23	13.0190	12.2323
22 → 23	12.5263	9.1583			

**Table 3:** Paths and their attributes.

Order	Handler and Zang's method			Proposed method		
	*k* shortest path	Delay	Cost	Important path	Delay	Cost
1	1-4-11-17-20-23	54.1798	48.7660	1-4-11-17-20-23	54.1798	48.7660
2	1-3-8-13-19-22-23	54.1798	48.7660	1-3-8-13-19-15-18-23	56.3484	78.8668
3	1-4-11-17-21-23	57.8418	55.6264	1-4-7-10-17-21-23	64.6681	69.9845
4	1-4-11-14-21-23	58.1237	61.0144	1-2-6-9-16-20-23	64.6681	69.9845
5	1-4-7-10-16-20-23	52.0383	61.7379	1-3-8-13-19-22-23	44.0553	74.5885
6	1-4-7-10-17-20-23	61.0060	63.1241			
7	1-4-7-11-17-20-23	61.3472	63.2659			
8	1-5-12-15-18-23	49.6080	64.2027			
9	1-5-11-17-20-23	54.6178	66.0392			
10	1-5-12-14-21-23	51.9017	69.8593			
11	1-5-8-13-19-22-23	48.6006	71.7413			
12	1-3-8-13-19-22-23	44.0553	74.5885			

**Table 4:** Setting parameter values.

BA model			Constraint	Cost		Delay		Proposed method	
*N*_0_	*E*_0_	*m*	*p*	Mean	std	Mean	std	*κ*	*γ*
3	3	3	0.1	10	3	10	5	2	30

**Table 5:** Computational results.

Nodes	Handler and Zang's method [14]	Proposed method
	Av. computer time (s)	Av. computer time (s)
100		
1	0.0147	0.236
2	0.0513	0.048
3	0.00559	0.0363
4	0.00575	0.0259
Av. time for 100 nodes	** 0.0193 **	**0.0866**
300		
1	0.0163	0.353
2	0.0189	1.009
3	0.0229	1.171
4	0.0148	0.231
Av. time for 300 nodes	** 0.0182 **	**0.691**
500		
1	0.038	1.278
2	0.037	1.14
3	0.039	1.298
4	0.039	1.346
Av. time for 500 nodes	** 0.0383**	** 1.266**
700		
1	0.733	3.989
2	0.0689	4.107
3	0.0791	5.563
4	0.069	3.775
Av. time for 700 nodes	** 0.238**	** 4.359**
1000		
1	0.121	17.34
2	0.126	24.531
3	0.143	20.135
4	0.135	42.846
Av. time for 1000 nodes	** 0.13125**	** 26.213**

Global Av.	0.0890	6.523
